# Assessing the Relationship Between Volumetric Changes and Functional Connectivity in Patients with Mild Cognitive Impairment

**DOI:** 10.3390/jcm15093229

**Published:** 2026-04-23

**Authors:** Weronika Machaj, Przemyslaw Podgorski, Julian Maciaszek, Dorota Szczesniak, Joanna Rymaszewska, Patryk Piotrowski, Anna Zimny

**Affiliations:** 1 Department of General and Interventional Radiology and Neuroradiology, Wroclaw Medical University, Borowska 213, 50-556 Wroclaw, Poland; przemyslaw.podgorski@umw.edu.pl (P.P.); anna.zimny@umw.edu.pl (A.Z.); 2 Department of General and Interventional Radiology and Neuroradiology, Wroclaw Medical University Hospital, Borowska 213, 50-556 Wroclaw, Poland; 3 Department of Psychiatry, Wroclaw Medical University, Pasteura 10, 50-367 Wroclaw, Poland; julian.maciaszek@umw.edu.pl (J.M.); dorota.szczesniak@umw.edu.pl (D.S.); patryk.piotrowski@umw.edu.pl (P.P.); 4 Department of Clinical Neuroscience, Faculty of Medicine, Wroclaw University of Science and Technology, Hoene-Wrońskiego 13c, 50-372 Wroclaw, Poland; joanna.rymaszewska@pwr.edu.pl

**Keywords:** resting-state functional MRI, functional connectivity, brain volumetry, amnestic mild cognitive impairment

## Abstract

**Background:** Amnestic mild cognitive impairment (aMCI) is considered a transitional state between normal aging and dementia, often without visible abnormalities on standard brain magnetic resonance (MR) images. The aim of the study was to analyze both microstructural and functional brain abnormalities using advanced MR techniques. **Methods**: The study included 27 patients with aMCI and an age-matched control group (CG) of 25 healthy subjects. All MR studies were performed on a 3T MR scanner (Philips, Ingenia) with a 32-channel head and neck coil using volumetric 3D T1 sequences, followed by a resting-state functional MRI (rs-fMRI) sequence. Volumetric analysis was performed using the Destrieux atlas to assess potential structural differences between groups. Seed-to-voxel functional connectivity analyses were conducted using the bilateral hippocampi and both anterior and posterior divisions of the parahippocampal gyri as seed regions. **Results:** Compared to healthy controls, reduced cortical thickness was observed in aMCI subjects in the temporal regions, frontal and orbitofrontal areas, limbic areas, parietal and sensorimotor cortices, as well as occipito-temporal regions. Additionally, significantly increased functional connectivity was observed between bilateral medial temporal lobe (MTL) regions and the right thalamus. **Conclusions:** Cortical thinning in various brain regions along with the increased functional connectivity between the MTL regions and the right thalamus may reflect potential compensatory mechanisms in response to initial subtle degenerative changes, emphasizing the importance of using both functional and structural imaging to detect early changes in aMCI patients.

## 1. Introduction

Mild cognitive impairment (MCI) is regarded as an early stage of cognitive decline, commonly involving memory or other cognitive functions, in individuals who remain functionally independent in everyday activities. It is widely considered an intermediary stage between normal aging and the development of dementia, especially aMCI, which is regarded as a precursor to Alzheimer’s disease (AD) [1,2]. This condition can be further classified into single-domain (S-aMCI), in which only memory is impaired, and multiple-domain (M-aMCI), where additional cognitive domains are affected. In the present study, we focused primarily on individuals with single-domain aMCI to obtain a more homogeneous group and to specifically investigate early memory-related alterations associated with prodromal Alzheimer’s disease [3].

One of the first symptoms in aMCI is difficulty remembering everyday information, such as recent conversations, personal experiences, or newly acquired knowledge, commonly referred to as everyday memory [1]. These early deficits may reduce quality of life and are associated with a higher prevalence of neuropsychiatric symptoms, including depression, apathy, and irritability, compared with cognitively healthy older adults [4,5].

The diagnosis of MCI is challenging and is based on reported cognitive concerns, objective evidence of impairment in one or more cognitive domains, preserved independence in daily functioning, and the absence of dementia [1,2,6]. Clinical evaluation includes patient history (often supplemented by informant input) and cognitive screening. The latter typically involves standard cognitive assessment tools (e.g., Montreal Cognitive Assessment (MoCA), Clinical Dementia Rating (CDR), Mini-Mental State Examination (MMSE)) and, when necessary, additional tools to assess symptoms of depression and anxiety, which frequently co-occur with early cognitive impairment and may influence cognitive performance [7,8,9,10].

Neuroimaging plays an important role in the evaluation of individuals with MCI by excluding structural causes of cognitive impairment, such as brain tumors, hematomas, or hydrocephalus. In standard MR examinations, patients with MCI usually show no overt abnormalities. The application of advanced neuroimaging techniques provides a unique opportunity to reveal microstructural or functional abnormalities within otherwise normal-appearing brains. Volumetric structural MRI studies have shown that individuals with MCI may exhibit subtle reductions in medial temporal lobe (MTL) regions, including the hippocampus and parahippocampal gyrus, as well as in the posterior cingulate cortex and precuneus [11,12]. These changes often precede widespread cortical atrophy and may serve as early indicators of neurodegenerative processes. Hippocampal atrophy, in particular, is one of the strongest predictors of progression to AD [13].

In addition to structural alterations, functional MRI (fMRI) can identify disturbances in brain connectivity in MCI. Resting-state fMRI (rs-fMRI) records spontaneous fluctuations in the blood oxygen level-dependent (BOLD) signal while the participant is not performing any specific task. These signal fluctuations reflect coordinated neuronal activity, allowing the assessment of temporal synchronization between distinct brain regions. As a result, rs-fMRI provides insight into the functional organization of large-scale brain networks and is well suited for detecting subtle connectivity changes that may precede overt structural degeneration [14]. Studies have consistently shown that individuals with MCI exhibit functional connectivity abnormalities, particularly in memory-related areas of the MTL, such as the hippocampus and parahippocampal gyrus [15,16]. Functional changes may occur independently of measurable volumetric atrophy, although they are often accompanied by slight cortical thinning in regions such as the superior temporal sulcus and cingulate cortex [17].

While previous research has predominantly examined either hippocampal atrophy or disruptions within large-scale networks such as the default mode network, relatively few studies have combined structural volumetric analysis with seed-based functional connectivity focused specifically on MTL regions [18,19]. Furthermore, the relationship between early structural changes and functional network reorganization in aMCI remains insufficiently understood, particularly in clinically homogeneous cohorts.

The aim of our study was to assess volumetric parameters of brain structures and resting-state functional connectivity in individuals with aMCI, with a particular focus on the MTL and its interactions with other brain regions. We hypothesized that individuals with aMCI would exhibit both reduced volumes of key MTL structures and altered functional connectivity within memory-related brain networks compared with cognitively healthy controls. Accordingly, the null hypothesis assumes no significant structural or functional differences between groups, whereas the alternative hypothesis assumes the presence of such alterations.

## 2. Materials and Methods

### 2.1. Study Participants

The study included 27 patients with clinically diagnosed aMCI and 25 healthy controls, matched for age, sex and education level (Table 1). Participants were recruited between January 2020 and December 2022 at the Department of Psychiatry, Wroclaw Medical University, Poland, through media advertisements and community-based outreach.

Eligibility was determined based on the following inclusion criteria: (A) the absence of other psychiatric conditions, such as depressive or anxiety disorders, that could influence cognitive performance, as assessed by the 15-item Geriatric Depression Scale (GDS-15) and the 14-item Hamilton Anxiety Rating Scale (HAM-A14); (B) a diagnosis of MCI according to the Petersen criteria, including (a) subjective memory complaints lasting at least 1–2 years, (b) objective memory deterioration confirmed by the MoCA, with a score between 19 and 26, (c) generally preserved global cognitive function assessed during the clinical interview, and (d) minimal impairment in daily activities based on the same interview; (C) age between 55 and 80 years; and (D) provision of written informed consent.

Participants were excluded from the study if they had any contraindications to MRI, such as claustrophobia, the presence of magnetic or ferromagnetic implants (electronic or mechanical) located in the head or neck region, or any brain pathology visible on MRI, including vascular lesions.

The diagnostic process involved a two-stage clinical assessment. Initially, participants underwent a cognitive evaluation performed by a trained psychologist. Subsequently, the diagnosis of aMCI and verification of inclusion and exclusion criteria were confirmed by a psychiatrist based on a clinical interview and standardized assessment tools.

MRI scans were independently reviewed by an experienced neuroradiologist to exclude structural brain abnormalities, including vascular lesions and other pathologies that could affect cognitive function.

No formal a priori sample size calculation was performed. The sample size was determined based on feasibility, including the availability of participants meeting strict inclusion criteria and the resource-intensive nature of MRI data acquisition and analysis. However, the final sample size is consistent with those reported in previous neuroimaging studies of aMCI [20,21].

This study was conducted in accordance with the Declaration of Helsinki and approved by the Bioethical Committee of Wroclaw Medical University (approval no. KB-400/2018/2506; approved on 25 June 2018).

### 2.2. Neuroimaging

Neuroimaging data were acquired during a 15 min session using a 3T MRI scanner (Ingenia, Philips, Best, The Netherlands) equipped with gradients of up to 45 mT/m and a maximum slew rate of 200 T/m/s, along with a 32-channel head coil. The imaging protocol included a high-resolution 3D T1-weighted sequence in the sagittal plane (257 sections; repetition time [TR] = 11 ms; echo time [TE] = 5 ms; flip angle = 8°; field of view [FOV] = 256 × 256 mm; voxel size = 0.75 × 0.75 × 0.75 mm^3^), followed by a 3D FLAIR sequence and a multiband echo-planar imaging (EPI) resting-state fMRI (rs-fMRI) sequence (multiband factor = 6; TR/TE = 1100/31 ms; voxel size = 2.5 × 2.5 × 2.5 mm^3^). To minimize head movement, foam padding was used, and participants wore headphones that reduced scanner noise. All subjects were instructed to remain awake with their eyes closed and not engage in any specific mental task.

3D FLAIR images were assessed visually by experienced neuroradiologists to exclude subjects with any brain pathology, particularly vascular lesions. 

Structural 3D T1 images and functional MRI data were preprocessed and analyzed using the CONN toolbox version 20.b, implemented in the MATLAB environment (R2019b; The MathWorks, Inc., Natick, MA, USA) and running in combination with SPM12 (Wellcome Department of Cognitive Neurology, University College London, London, UK) on a Linux-based HPC system (Ubuntu 18.04).

The preprocessing pipeline followed standard procedures and included the following steps: slice timing correction, realignment and unwarping, normalization to MNI space, segmentation, and spatial smoothing. Functional scans were first corrected for slice timing differences, and motion-related distortions were addressed through realignment and unwarping in SPM12 (Wellcome Department of Cognitive Neurology, University College London, London, UK) using b-spline interpolation, following standard preprocessing procedures widely used in contemporary neuroimaging pipelines [22]. Each scan was registered to a reference volume to ensure temporal consistency across runs. To detect motion artifacts and intensity outliers, the Artifact Detection Tools (ART) were applied. Scans were flagged as outliers if they exhibited signal fluctuations that exceeded ±3 standard deviations from the global mean BOLD signal or if framewise displacement surpassed 0.5 mm. These thresholds were selected in accordance with commonly used criteria in rs-fMRI preprocessing and are consistent with recommendations implemented in the CONN toolbox, allowing reliable detection of motion-related artifacts while preserving data quality [23]. Both anatomical and functional datasets were normalized to the Montreal Neurological Institute (MNI) standard space. Structural T1-weighted images were used for anatomical referencing, and the mean functional image served as the reference for aligning rs-fMRI volumes. Segmentation of anatomical images into gray matter, white matter, and cerebrospinal fluid (CSF) was performed using SPM12’s unified segmentation algorithm.

The normalized data were resampled to isotropic voxels: 1 mm for anatomical and 2 mm for functional scans, using fourth-order spline interpolation. The final bounding box dimensions were set to 180 × 216 × 180 mm. 

To improve data quality and minimize confounding factors in the resting-state BOLD signal, several denoising strategies were implemented. These included removal of signal components from CSF and white matter regions, correction for head motion using six motion parameters (translation and rotation), and their first-order temporal derivatives. Additionally, the CompCor method was used to further isolate and regress out physiological noise components [23,24].

Functional data were subjected to temporal band-pass filtering in the 0.008–0.09 Hz frequency range using discrete cosine transform (DCT) basis sets. This step helps isolate slow-frequency fluctuations associated with resting-state neural activity while reducing high-frequency physiological noise and low-frequency drift [25]. Finally, to increase the signal-to-noise ratio and reduce inter-subject anatomical variability, spatial smoothing was applied to the functional data using an 8 mm full-width at half-maximum (FWHM) Gaussian kernel [22].

#### 2.2.1. Volumetric Analysis

Structural T1-weighted images were processed using the CAT12 (Structural Brain Mapping Group, Jena University Hospital, Jena, Germany) toolbox. ROI-based gray matter volumetric measures were extracted with the CAT12 ROIs pipeline using the Neuromorphometrics atlas in combination with the CoBrA atlas for a more detailed characterization of limbic subregions. Cortical thickness was quantified according to the Destrieux atlas (aparc.a2009s), allowing fine-grained gyral and sulcal segmentation.

#### 2.2.2. Seed-to-Voxel Functional Connectivity Analysis

Resting-state functional connectivity was examined using a seed-to-voxel approach implemented in the CONN toolbox. Bilateral hippocampi and the anterior and posterior divisions of the bilateral parahippocampal gyri were defined as regions of interest (ROIs) based on the Human Connectome Project (HCP) atlas. These ROIs were selected because they comprise key MTL structures involved in memory processing and are particularly relevant to early cognitive decline.

For each participant, seed-based connectivity maps were computed as Fisher-transformed bivariate correlation coefficients between the mean BOLD time series extracted from each ROI and the BOLD time series of every voxel in the brain. Individual connectivity maps were then entered into second-level analyses to assess between-group differences in functional connectivity.

Group effects were evaluated within the general linear model framework using voxel-wise two-sample t-contrasts comparing participants with aMCI and controls, while including age, sex, and mean framewise displacement as covariates of no interest. Statistical significance was assessed using a voxel-level height threshold of *p* < 0.001 (uncorrected) and a cluster-level extent threshold of *p* < 0.05, family-wise error (FWE) corrected for multiple comparisons. Exact *p*-values for significant effects are reported in the Section 3.

## 3. Results

No volumetric differences survived correction for multiple comparisons in regional gray matter volumes derived from CAT12 (Supplementary Tables S1 and S2). However, several regions, particularly within limbic and cerebellar structures, showed nominally significant (uncorrected) group differences.

**Figure 1:**
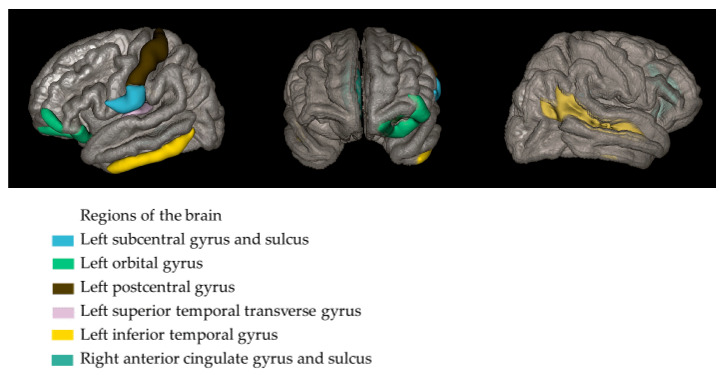
Brain regions showing significant cortical thinning in the aMCI group compared with controls, based on cortical thickness analysis using the Destrieux atlas. Colored areas indicate regions with statistically significant differences, with colors corresponding to anatomical regions.

In contrast, cortical thickness analysis using the Destrieux atlas (aparc.a2009s) showed significant thinning in the aMCI group compared with the CG in the following regions: the right anterior cingulate gyrus and sulcus, the right superior temporal sulcus, the left transverse temporal gyrus, the left inferior temporal gyrus, the left orbital gyrus, the left H-shaped orbital sulcus, the left subcentral gyrus and the sulcus, the left postcentral gyrus, and the medial occipito-temporal and the lingual sulcus (*p*-values ranging from *p* = 0.00004 to *p* = 0.00029; Figure 1, Table 2).

**Figure 2:**
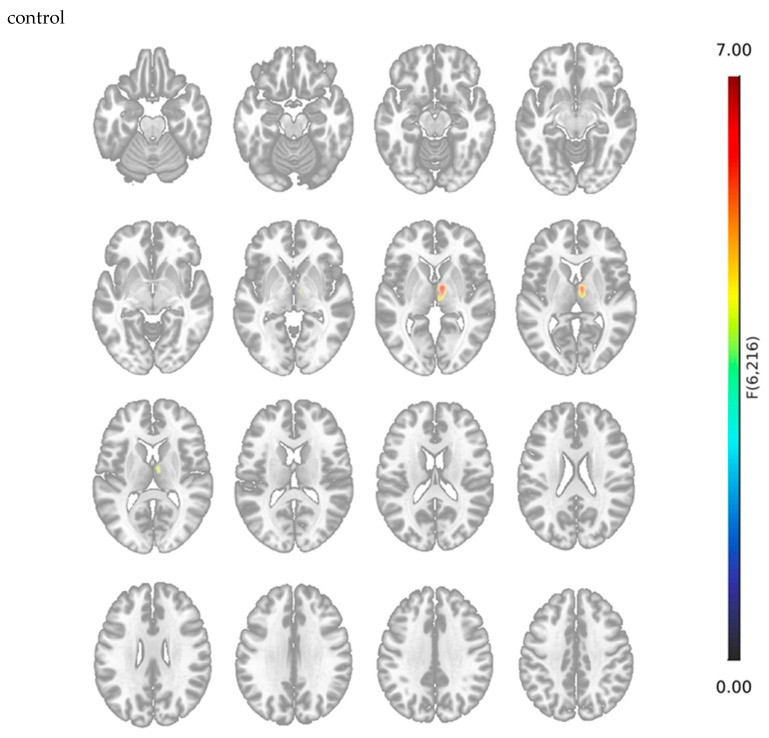
Seed-to-voxel resting-state functional connectivity analysis using the bilateral MTL regions (hippocampi and anterior/posterior parahippocampal gyri). The figure shows a statistical map of significantly increased functional connectivity with the right thalamus (marked in red) in individuals with aMCI compared to CG, overlaid on a standard anatomical brain template. The results indicate significantly increased functional connectivity with the right thalamus (aMCI > CG; pFDR = 0.031).

Seed-to-voxel resting-state functional connectivity analysis was performed using bilateral hippocampi and the anterior and posterior divisions of the parahippocampal gyri as seed regions. Compared to cognitively healthy controls, individuals with aMCI showed significantly increased functional connectivity between the seed regions and the right thalamus (aMCI > CG; pFDR = 0.031; Figure 2).

## 4. Discussion

The main objective of this study was to analyze changes in brain microstructure and in resting-state functional connectivity in individuals with aMCI with a particular focus on the MTL and its interaction with other brain regions. Using a combination of volumetric assessment and seed-to-voxel functional connectivity analysis, we aimed to examine both the microstructural and functional correlates of early cognitive decline, including subtle abnormalities that may remain undetectable in standard structural MRI examinations of individuals with MCI.

The volumetric analysis did not reveal statistically significant differences after correction for multiple comparisons in regional gray matter volumes between the aMCI and CG. However, several regions showed nominally significant (uncorrected) group differences. A possible explanation for the absence of corrected volumetric differences is that early or subtle neurodegenerative changes characteristic of aMCI may manifest primarily as cortical thinning rather than overt volume loss, which is typically detectable only at more advanced disease stages. In line with this interpretation, cortical thickness analysis using the Destrieux atlas revealed a widespread pattern of cortical thinning. The affected regions included temporal areas (left superior transverse temporal gyrus, left inferior temporal gyrus, right superior temporal sulcus), frontal and orbitofrontal regions (left orbital gyrus, left H-shaped orbital sulcus), limbic areas (right anterior cingulate gyrus and sulcus), parietal and sensorimotor cortices (left subcentral gyrus and sulcus, left postcentral gyrus), as well as occipito-temporal regions (left medial occipito-temporal and lingual sulcus).

Among these regions, the right superior temporal sulcus (STS) is particularly notable. The STS, which separates the superior from the middle temporal gyrus, is known to play a central role in processing biological motion, voice, dynamic aspects of faces, and higher-order social cognition such as theory of mind [26]. Although direct evidence for cortical thinning of the STS in aMCI is more limited, converging data indicate that neurodegeneration in aMCI and AD extends beyond the MTL and superior temporal gyrus. Longitudinal studies report sequential atrophic changes within temporal regions (with some evidence also implicating the STS), suggesting that sulcal structures such as the STS may also undergo early degeneration during cognitive decline [27,28].

We also found cortical thinning in the superior transverse temporal gyrus, the primary auditory cortex [29]. Studies have demonstrated morphological alterations in the transverse temporal gyrus in aMCI compared to individuals with subjective cognitive decline, indicating early involvement of auditory processing regions [30]. Moreover, age-related hearing loss has been associated with reductions in cortical thickness in auditory and related brain regions, including parts of the superior temporal cortex such as the transverse temporal gyrus, and these structural changes may contribute to cognitive decline in conditions like aMCI [31].

Together, these findings suggest the notion that auditory and language-related cortical regions are particularly vulnerable in aMCI, and that their degeneration may contribute to both cognitive and sensory decline in the early stages of the disease.

Additional thinning was observed in the left inferior temporal gyrus, an area consistently associated with semantic memory, visual object recognition, and higher-order perceptual processing [32]. Its involvement in aMCI may reflect a broader pattern of early neurodegeneration extending beyond auditory and language networks. Consistent with this interpretation, in the Alzheimer’s Disease Neuroimaging Initiative (ADNI) cohort, cortical thinning of the inferior temporal gyrus has been observed in individuals with late MCI in association with increased amyloid burden [33]. Reduced cortical volumes in inferior temporal regions among aMCI patients have also been reported by Whitwell et al. [34].

The left orbital gyrus and H-shaped orbital sulcus, which are important components of the orbitofrontal cortex, play a significant role in stimulus evaluation, inhibitory control, and socio-emotional regulation [35]. The thinning observed in these regions in our study has also been reported in the literature. For example, Zhao et al. demonstrated reduced cortical thickness in the left lateral orbitofrontal gyrus in both MCI and AD patients compared with cognitively normal individuals, with more pronounced thinning in AD, indicating early involvement of orbitofrontal subregions in cognitive impairment [36].

The right anterior cingulate gyrus and sulcus, parts of the anterior cingulate cortex (ACC), are involved in emotion regulation, conflict monitoring, inhibitory control, and adaptive behavior [37]. Jeong et al. reported significantly reduced cortical thickness of the anterior cingulate cortex in patients with aMCI, with greater thinning predicting conversion to AD, particularly in individuals with psychotic symptoms [38]. Similarly, Gonzales et al. showed that cortical atrophy involving the anterior cingulate region was associated with accelerated cognitive decline in MCI patients with subsyndromal depression [39].

The postcentral gyrus (primary somatosensory cortex) and the adjacent subcentral region play key roles in sensory processing and sensorimotor integration [40]. In surface-based morphometry (SBM) analysis, cortical thickness in MCI patients compared with individuals with normal cognition showed significant thinning in multiple brain regions, including the bilateral postcentral gyrus, suggesting that disrupted sensorimotor integration may contribute to visuospatial and psychomotor difficulties [41].

Finally, cortical thinning in occipito-temporal regions, including the left medial occipito-temporal and lingual sulcus, is consistent with previous evidence linking these areas to visual processing, object recognition, and visuospatial integration [42]. Seo et al. reported widespread cortical thinning in MCI patients with small-vessel disease, including prominent thinning of the lingual gyrus, together with alterations in frontal, cingulate, insular, and superior temporal regions [43]. Cheng et al. reported occipito-temporal cortical thinning in individuals with MCI compared with healthy controls, with cortical thickness in these regions showing significant associations with neuropsychological performance [28]. 

In addition to these structural alterations, we also observed functional changes. Resting-state analysis revealed significantly increased connectivity between structures in the MTL and the right thalamus in aMCI, a pattern that has been reported only in a limited number of previous rs-fMRI studies of MCI, with heterogeneous findings.

The hippocampus is essential for the encoding and consolidation of episodic memories. It also binds spatial and contextual elements into coherent representations [44]. In MCI, functional changes in the hippocampus may reflect potential network-level adaptations or early functional reorganization, rather than strictly compensatory mechanisms. Closely interconnected with the hippocampus, the parahippocampal cortex is involved in processing spatial layouts and contextual cues, serving as a critical interface for relaying multimodal sensory and associative inputs to the hippocampus [45]. Altered functional connectivity of this region in MCI may indicate a shift in network dynamics or functional reorganization as memory efficiency begins to decline. The thalamus coordinates the transfer of information between cortical and subcortical structures and contributes to attention, working memory, and temporal coordination of neural activity [46]. Due to their key role in memory and network integration, the hippocampus, parahippocampal cortex, and thalamus are considered key structures in the pathomechanism of MCI.

Only a limited number of rs-fMRI studies have reported impaired functional connectivity between structures in the MTL and the thalamus in MCI.

Most of the available evidence points to preserved or reduced functional connectivity between the thalamus and the MTL, especially in more advanced stages of the disease.

For example, using ADNI data, Cai et al. demonstrated that in late-stage aMCI, functional connectivity between the right thalamus and the hippocampus was significantly reduced compared with early MCI, with no regions showing increased thalamo-hippocampal connectivity [47].

In contrast, increased hippocampus-thalamus connectivity has been reported in AD. Specifically, Xue et al. showed that patients with AD exhibited increased functional connectivity between the left hippocampus and the right thalamus compared with individuals with MCI in a resting-state seed-based analysis [48].

In summary, these findings suggest the possibility of a nonlinear trajectory of functional connectivity between the thalamus and the MTL over the course of AD progression. The increased connectivity observed in aMCI in this study may reflect an early network-level response, which could be related to early network reorganization or altered functional dynamics, although this interpretation remains speculative and requires confirmation in longitudinal studies. As the disease progresses, these connections appear to weaken, possibly reflecting emerging network disturbances. In more advanced stages, such as AD, increased connectivity between the thalamus and hippocampus has again been reported, which is more likely to reflect abnormal network synchronization or loss of inhibitory control rather than effective compensation. This pattern suggests that changes in connectivity between the thalamus and the MTL are dynamic and stage-dependent, and increased functional connectivity should not be uniformly interpreted as beneficial or pathological without considering the stage of the disease.

Overall, our results indicate that functional and structural changes may occur in the early stages of aMCI, reflecting the multiple aspects of emerging neurodegenerative processes. Although volumetric measurements did not reveal significant differences between groups, analyses of cortical thickness and functional connectivity were more sensitive to early changes, highlighting the value of combining rs-fMRI with high-resolution morphometric methods. Combining these methods may improve the early detection of subtle changes in the brain associated with cognitive decline.

## 5. Strengths and Limitations

The rs-fMRI post-processing method used in our study is a global, standardized tool. This approach ensures that our methods are reproducible and that our results can be easily compared in future studies. Moreover, the combination of functional connectivity analysis and volumetric assessment allows for a comprehensive evaluation of both structural and functional brain changes in aMCI.

However, there are some limitations to consider. Our study included a relatively small sample size, which may limit the generalizability of our findings. The sample size was determined based on feasibility considerations, including the availability of participants meeting strict inclusion criteria and the resource-intensive nature of MRI acquisition and analysis. Future studies with larger cohorts and more detailed stratification of cognitive impairment are needed to validate and extend our results.

Another limitation of our study is that we included only patients with the amnestic subtype of MCI. Although this subtype is the most commonly associated with progression to AD, restricting the sample to aMCI limits the generalizability of our findings to other MCI subtypes. Non-amnestic forms may present with different patterns of structural and functional brain alterations, and therefore future research should incorporate a broader range of MCI phenotypes to allow for more comprehensive comparisons.

In addition, the cross-sectional design of the study limits the ability to draw conclusions about the temporal progression of the observed changes and does not allow for direct assessment of longitudinal trajectories.

Another limitation is the lack of direct analysis linking imaging findings with cognitive performance measures, which restricts the interpretation of the clinical relevance of the observed structural and functional alterations.

Finally, the single-center design of the study may introduce potential site- or scanner-specific bias, which could further limit the generalizability of the results.

## 6. Conclusions

Our findings suggest that aMCI may be associated with diffuse thinning of the cerebral cortex, even in the absence of measurable differences in regional gray matter volumes. This indicates that cortical thickness measures may be more sensitive than volumetric indices for detecting early structural changes. Importantly, despite these subtle structural changes, we observed increased resting-state functional connectivity between MTL structures and the right thalamus, which may reflect early network-level reorganization. Such functional hyperconnectivity could represent a transient adaptive response, potentially offsetting the impact of cortical thinning. Overall, these results highlight the potential complementary value of multimodal neuroimaging approaches in capturing both the anatomical and functional dynamics underlying early stages of aMCI.

## Figures and Tables

**Table 1:** Demographic and clinical information on the study group.

Demographic Data	aMCI	Control Group (CG)	Cohen’s d
Participants (*n*)	27	25	-
Age (years, mean)	70.1	65.7	-
Female/male (*n*)	13/14	13/12	-
Education (years, mean)	15.1	15.5	-
MoCA (points, mean ± SD)	24.4 (1.87)	27.3 (1.2)	−1.83
CDR (score)	0.5	0	-
DemTect (points, mean ± SD)	11.82 (1.66)	13.36 (1.32)	−1.02
GDS-15 (points, mean ± SD)	4.14 (1.18)	1.3 (1)	2.59
HAM-A 14 (points, mean ± SD)	5.36 (2.93)	3.2 (2.1)	0.84

Abbreviations and score ranges: MoCA—Montreal Assessment Cognitive Scale (0–30 points; lower scores indicate worse cognitive performance; scores < 25 suggest cognitive impairment); CDR—Clinical Dementia Rating Scale (0 = normal cognition, 0.5 = mild cognitive impairment); GDS-15—Geriatric Depression Scale (0–15 points; ≥5 suggests clinically relevant depressive symptoms); DemTect—Dementia Detection Test (0–18 points; scores ≤ 12 suggest cognitive impairment); HAM-A 14—Hamilton Anxiety Rating Scale (0–56 points; higher scores indicate greater anxiety severity; scores < 7 are considered within the normal range).

**Table 2:** Statistically significant results of cortical thickness (in mm) using the Destrieux atlas.

Brain Regions	Cortical Thickness (mm, Mean)		*p*-Value
	aMCI	CG	
Right anterior cingulate gyrus and sulcus	2.283	2.408	0.00021
Right superior temporal sulcus	2.290	2.436	0.00005
Left superior temporal transverse gyrus	2.037	2.296	0.00004
Left inferior temporal gyrus	2.210	2.403	0.00029
Left orbital gyrus	2.250	2.397	0.00022
Left H-shaped orbital sulcus	2.213	2.342	0.00024
Left subcentral gyrus and sulcus	2.316	2.486	0.00008
Left postcentral gyrus	1.827	2.041	0.00004
Left medial occipito-temporal and lingual sulcus	2.163	2.339	0.00024

## Data Availability

The original contributions presented in this study are included in the article/Supplementary Material. Further inquiries can be directed to the corresponding author(s).

## Supplementary Materials

The following supporting information can be downloaded at: https://www.mdpi.com/article/10.3390/jcm15093229/s1, Table S1. Regional gray matter volumes derived from CAT12 using the CoBrA. Values are presented as mean volumes (cm^3^); reported p-values are uncorrected for multiple comparisons. Table S2. Regional gray matter volumes derived from CAT12 based on the Desikan–Killiany (aparc DK40) cortical atlas. Values are presented as mean volumes (cm^3^); reported p-values are uncorrected for multiple comparisons.
