# Supplementary material for: Assessing the Relationship Between Volumetric Changes and Functional Connectivity in Patients with Mild Cognitive Impairment
Source: J Clin Med. 2026 Apr 23;15(9):3229. doi: 10.3390/jcm15093229 (PMC13164081; doi:10.3390/jcm15093229)
Supplement: Supplementary file 1 [file jcm-15-03229-s001.zip › jcm-4229953-supplementary.pdf]

Table S1. Regional gray matter volumes derived from CAT12 using the CoBrA. Values are presented as mean volumes (cm³); reported p-values are uncorrected for multiple comparisons.

**Brain regions**	**Regional gray matter volumes**				**p-value**
	**aMCI (cm³, mean ± SD)**	**aMCI (SE)**	**CG (cm³, mean ± SD)**	**CG (SE)**	
Left striatum	8.00 (0.93)	0.18	8.66 (1.03)	0.21	0.050
Left globus pallidus	0.36 (0.14)	0.03	0.36 (0.10)	0.02	0.450
Left thalamus	4.57 (0.50)	0.10	4.75 (0.47)	0.09	0.128
Left anterior cerebellum (lobules I–II)	0.07 (0.01)	0.00	0.07 (0.01)	0.00	0.049
Left anterior cerebellum (lobule III)	0.64 (0.08)	0.02	0.71 (0.07)	0.01	0.007
Left anterior cerebellum (lobule IV)	1.70 (0.20)	0.04	1.84 (0.17)	0.03	0.011
Left anterior cerebellum (lobule V)	3.05 (0.35)	0.07	3.32 (0.39)	0.08	0.018
Left superior posterior cerebellum (lobule VI)	6.25 (0.79)	0.15	6.67 (0.77)	0.15	0.057
Left superior posterior cerebellum (Crus I)	8.75 (1.29)	0.25	9.61 (1.33)	0.27	0.027
Left superior posterior cerebellum (Crus II)	5.57 (0.72)	0.14	6.46 (0.76)	0.15	< 0.001
Left superior posterior cerebellum (lobule VIIB)	2.95 (0.28)	0.05	3.47 (0.38)	0.08	< 0.001
Left inferior posterior cerebellum (lobule VIIIA)	4.41 (0.37)	0.07	5.00 (0.47)	0.09	< 0.001
Left inferior posterior cerebellum (lobule VIIIB)	2.66 (0.36)	0.07	2.82 (0.34)	0.07	0.086
Left inferior posterior cerebellum (lobule IX)	2.38 (0.33)	0.06	2.59 (0.32)	0.06	0.027

Left inferior posterior cerebellum (lobule X)	0.52 (0.05)	0.01	0.56 (0.06)	0.01	0.011
Left amygdala	1.43 (0.19)	0.04	1.58 (0.23)	0.05	0.017
Left hippocampus (CA1 subfield)	0.91 (0.10)	0.02	1.03 (0.13)	0.03	0.002
Left subiculum	0.49 (0.06)	0.01	0.52 (0.06)	0.01	0.051
Left fornix	0.25 (0.04)	0.01	0.28 (0.04)	0.01	0.024
Left hippocampus (CA4 subfield)	0.64 (0.09)	0.02	0.74 (0.01)	0.02	0.001
Left hippocampus (CA2/CA3 subfields)	0.23 (0.06)	0.01	0.27 (0.05)	0.01	0.017
Left hippocampal stratum radiatum/lacunosum-moleculare	0.34 (0.04)	0.01	0.38 (0.04)	0.01	0.005
Left fimbria of hippocampus	0.09 (0.02)	0.00	0.11 (0.11)	0.00	0.005
Left mammillary body	0.01 (0.01)	0.00	0.01 (0.01)	0.00	0.096
Left alveus of hippocampus	0.15 (0.06)	0.01	0.19 (0.04)	0.01	0.015
Right striatum	8.23 (0.97)	0.19	8.93 (1.10)	0.22	0.023
Right globus pallidus	0.37 (0.13)	0.02	0.38 (0.12)	0.02	0.411
Right thalamus	4.43 (0.59)	0.11	4.83 (0.50)	0.10	0.016
Right anterior cerebellum (lobules I–II)	0.10 (0.02)	0.00	0.11 (0.03)	0.00	0.132
Right anterior cerebellum (lobule III)	0.71 (0.08)	0.02	0.76 (0.07)	0.01	0.014
Right anterior cerebellum (lobule IV)	1.48 (0.16)	0.03	1.55 (0.16)	0.03	0.075

Right anterior cerebellum (lobule V)	2.99 (0.32)	0.06	3.19 (0.36)	0.07	0.038
Right superior posterior cerebellum (lobule VI)	6.30 (0.81)	0.16	7.02 (1.00)	0.20	0.011
Right superior posterior cerebellum (Crus I)	9.01 (1.43)	0.28	9.63 (1.15)	0.23	0.077
Right superior posterior cerebellum (Crus II)	6.11 (0.85)	0.16	6.96 (0.63)	0.13	0.001
Right superior posterior cerebellum (lobule VIIB)	3.39 (0.44)	0.09	3.94 (0.36)	0.07	< 0.001
Right inferior posterior cerebellum (lobule VIIIA)	3.33 (0.44)	0.09	3.77 (0.49)	0.10	0.004
Right inferior posterior cerebellum (lobule VIIIB)	2.53 (0.35)	0.07	2.84 (0.42)	0.08	0.009
Right inferior posterior cerebellum (lobule IX)	2.24 (0.31)	0.06	2.46 (0.31)	0.06	0.019
Right inferior posterior cerebellum (lobule X)	0.52 (0.07)	0.01	0.55 (0.06)	0.01	0.060
Right amygdala	1.43 (0.21)	0.04	1.60 (0.18)	0.03	0.005
Right hippocampus (CA1 subfield)	1.01 (0.13)	0.02	1.11 (0.16)	0.02	0.008
Right subiculum	0.48 (0.06)	0.01	0.53 (0.11)	0.01	0.009
Right fornix	0.26 (0.05)	0.01	0.29 (0.04)	0.01	0.001
Right hippocampus (CA4 subfield)	0.67 (0.08)	0.02	0.76 (0.08)	0.02	0.001
Right hippocampus (CA2/CA3 subfields)	0.28 (0.05)	0.01	0.32 (0.04)	0.01	0.004
Right hippocampal stratum radiatum/lacunosum-moleculare	0.34 (0.04)	0.01	0.38 (0.03)	0.01	0.002
Right fimbria of hippocampus	0.08 (0.02)	0.00	0.09 (0.01)	0.00	0.071

Right mammillary body	0.01 (0.01)	0.00	0.02 (0.01)	0.00	0.071
Right alveus of hippocampus	0.14 (0.03)	0.01	0.17 (0.02)	0.00	0.004

Table S2. Regional gray matter volumes derived from CAT12 based on the Desikan–Killiany (aparc DK40) cortical atlas. Values are presented as mean volumes (cm^3); reported p-values are uncorrected for multiple comparisons.

**Brain regions**	**Regional gray matter volumes**				**p-value**
	**aMCI (cm^3, mean ± SD)**	**aMCI (SE)**	**CG (cm^3, mean ± SD)**	**CG (SE)**	
Left banks of the superior temporal sulcus	2.23 (0.10)	0.02	2.39 (0.12)	0.02	< 0.001
Right banks of the superior temporal sulcus	2.30 (0.11)	0.02	2.49 (0.12)	0.02	< 0.001
Left caudal anterior cingulate cortex	2.07 (0.23)	0.04	2.21 (0.20)	0.04	0.053
Right caudal anterior cingulate cortex	1.99 (0.19)	0.04	2.21 (0.22)	0.04	0.002
Left caudal middle frontal gyrus	2.40 (0.13)	0.03	2.47 (0.12)	0.02	0.091
Right caudal middle frontal gyrus	2.41 (0.13)	0.02	2.51 (0.11)	0.02	0.016
Left cuneus	1.79 (0.12)	0.02	1.89 (0.12)	0.02	0.018
Right cuneus	1.75 (0.13)	0.03	1.90 (0.11)	0.02	0.001
Left entorhinal cortex	2.36 (0.30)	0.06	2.64 (0.48)	0.10	0.037
Right entorhinal cortex	2.39 (0.35)	0.07	2.52 (0.37)	0.07	0.257
Left fusiform gyrus	2.27 (0.17)	0.03	2.45 (0.13)	0.03	0.001
Right fusiform gyrus	2.28 (0.11)	0.02	2.42 (0.13)	0.03	0.002
Left inferior parietal lobule	2.20 (0.10)	0.02	2.29 (0.11)	0.02	0.020
Right inferior parietal lobule	2.21 (0.09)	0.02	2.33 (0.10)	0.02	< 0.001
Left inferior temporal gyrus	2.21 (0.14)	0.03	2.38 (0.12)	0.02	0.001

Right inferior temporal gyrus	2.29 (0.12)	0.02	2.40 (0.14)	0.03	0.008
Left isthmus of the cingulate cortex	1.85 (0.13)	0.02	1.91 (0.11)	0.02	0.152
Right isthmus of the cingulate cortex	1.82 (0.09)	0.02	1.89 (0.13)	0.03	0.074
Left lateral occipital cortex	1.97 (0.18)	0.04	2.10 (0.11)	0.02	0.010
Right lateral occipital cortex	1.98 (0.11)	0.02	2.09 (0.10)	0.02	0.004
Left lateral orbitofrontal cortex	2.27 (0.10)	0.02	2.39 (0.09)	0.02	< 0.001
Right lateral orbitofrontal cortex	2.22 (0.10)	0.02	2.32 (0.13)	0.03	0.012
Left lingual gyrus	1.85 (0.12)	0.02	2.00 (0.14)	0.03	0.001
Right lingual gyrus	1.84 (0.14)	0.03	1.95 (0.14)	0.03	0.015
Left medial orbitofrontal cortex	1.95 (0.11)	0.02	2.05 (0.10)	0.02	0.005
Right medial orbitofrontal cortex	1.98 (0.09)	0.02	2.07 (0.10)	0.02	0.005
Left middle temporal gyrus	2.38 (0.14)	0.03	2.55 (0.17)	0.03	0.002
Right middle temporal gyrus	2.47 (0.13)	0.03	2.59 (0.13)	0.03	0.008
Left parahippocampal gyrus	2.20 (0.18)	0.04	2.36 (0.19)	0.04	0.015
Right parahippocampal gyrus	2.21 (0.13)	0.02	2.30 (0.15)	0.03	0.067
Left paracentral lobule	2.08 (0.18)	0.03	2.22 (0.13)	0.03	0.009
Right paracentral lobule	2.08 (0.17)	0.03	2.18 (0.19)	0.04	0.094
Left pars opercularis	2.43 (0.13)	0.02	2.47 (0.12)	0.02	0.307
Right pars opercularis	2.40 (0.09)	0.02	2.48 (0.11)	0.02	0.028
Left pars orbitalis	2.30 (0.10)	0.02	2.43 (0.11)	0.02	0.001
Right pars orbitalis	2.31 (0.13)	0.03	2.41 (0.12)	0.02	0.020

Left pars triangularis	2.26 (0.11)	0.02	2.34 (0.10)	0.02	0.022
Right pars triangularis	2.27 (0.12)	0.02	2.35 (0.11)	0.02	0.034
Left pericalcarine cortex	1.60 (0.17)	0.03	1.75 (0.15)	0.03	0.004
Right pericalcarine cortex	1.62 (0.23)	0.04	1.77 (0.17)	0.03	0.027
Left postcentral gyrus	1.86 (0.11)	0.02	2.02 (0.10)	0.02	< 0.001
Right postcentral gyrus	1.88 (0.11)	0.02	2.01 (0.12)	0.02	0.001
Left posterior cingulate cortex	2.02 (0.09)	0.02	2.07 (0.07)	0.01	0.044
Right posterior cingulate cortex	2.01 (0.10)	0.02	2.06 (0.10)	0.02	0.144
Left precentral gyrus	2.13 (0.13)	0.03	2.28 (0.16)	0.03	0.004
Right precentral gyrus	2.11 (0.18)	0.03	2.26 (0.13)	0.03	0.005
Left precuneus	2.17 (0.10)	0.02	2.24 (0.11)	0.02	0.037
Right precuneus	2.18 (0.10)	0.02	2.26 (0.11)	0.02	0.040
Left rostral anterior cingulate cortex	2.25 (0.12)	0.02	2.35 (0.11)	0.02	0.004
Right rostral anterior cingulate cortex	2.26 (0.12)	0.02	2.39 (0.16)	0.03	0.010
Left rostral middle frontal gyrus	2.18 (0.09)	0.02	2.27 (0.11)	0.02	0.008
Right rostral middle frontal gyrus	2.18 (0.10)	0.02	2.26 (0.11)	0.02	0.034
Left superior frontal gyrus	2.47 (0.11)	0.02	2.58 (0.12)	0.02	0.004
Right superior frontal gyrus	2.48 (0.11)	0.02	2.59 (0.11)	0.02	0.004
Left superior parietal lobule	2.07 (0.15)	0.03	2.14 (0.09)	0.02	0.075
Right superior parietal lobule	2.08 (0.12)	0.02	2.15 (0.10)	0.02	0.047
Left superior temporal gyrus	2.18 (0.16)	0.03	2.41 (0.15)	0.03	< 0.001

Right superior temporal gyrus	2.32 (0.16)	0.03	2.50 (0.15)	0.03	0.001
Left supramarginal gyrus	2.24 (0.09)	0.02	2.35 (0.10)	0.02	0.001
Right supramarginal gyrus	2.27 (0.09)	0.02	2.36 (0.10)	0.02	0.008
Left frontal pole	2.20 (0.19)	0.04	2.33 (0.18)	0.04	0.049
Right frontal pole	2.11 (0.14)	0.03	2.30 (0.15)	0.03	< 0.001
Left temporal pole	2.71 (0.20)	0.04	2.94 (0.48)	0.10	0.057
Right temporal pole	2.72 (0.27)	0.05	2.84 (0.46)	0.09	0.326
Left transverse temporal gyrus (Heschl's gyrus)	2.02 (0.18)	0.03	2.26 (0.14)	0.03	< 0.001
Right transverse temporal gyrus (Heschl's gyrus)	2.04 (0.22)	0.04	2.26 (0.14)	0.03	0.001
Left insular cortex	2.48 (0.15)	0.03	2.54 (0.19)	0.04	0.270
Right insular cortex	2.49 (0.14)	0.03	2.61 (0.15)	0.03	0.021
